# Magnetic resonance-guided focused ultrasound treatment of facet joint pain: summary of preclinical phase

**DOI:** 10.1186/2050-5736-2-9

**Published:** 2014-02-28

**Authors:** Sagi Harnof, Zion Zibly, Lilach Shay, Osnat Dogadkin, Arik Hanannel, Yael Inbar, Itay Goor-Aryeh, Israel Caspi

**Affiliations:** 1 Department of Neurological Surgery, Sheba Medical Center, Ramat Gan 52621, Israel; 2 Department of Neurological Surgery, The Ohio State University Medical Center, Columbus, OH 43210, USA; 3 The Focus Ultrasound Foundation, Charlottesville, VA 22903, USA; 4 Department of Radiology, Sheba Medical Center, Ramat Gan 52621, Israel; 5 Department of Anesthesiology, Sheba Medical Center, Ramat Gan 52621, Israel; 6 Department of Orthopedic Surgery, Sheba Medical Center, Ramat Gan 52621, Israel

**Keywords:** Chronic back pain, Facet joints, MRgFUS, Pain palliation

## Abstract

**Study design:**

A phantom experiment, two thermocouple experiments, three *in vivo* pig experiments, and a simulated treatment on a healthy human volunteer were conducted to test the feasibility, safety, and efficacy of magnetic resonance-guided focused ultrasound (MRgFUS) for treating facet joint pain.

**Objective:**

The goal of the current study was to develop a novel method for accurate and safe noninvasive facet joint ablation using MRgFUS.

**Summary of background data:**

Facet joints are a common source of chronic back pain. Direct facet joint interventions include medial branch nerve ablation and intra-articular injections, which are widely used, but limited in the short and long term. MRgFUS is a breakthrough technology that enables accurate delivery of high-intensity focused ultrasound energy to create a localized temperature rise for tissue ablation, using MR guidance for treatment planning and real-time feedback.

**Methods:**

We validated the feasibility, safety, and efficacy of MRgFUS for facet joint ablation using the ExAblate 2000® System (InSightec Ltd., Tirat Carmel, Israel) and confirmed the system's ability to ablate the edge of the facet joint and all terminal nerves innervating the joint. A phantom experiment, two thermocouple experiments, three in *vivo pig* experiments, and a simulated treatment on a healthy human volunteer were conducted.

**Results:**

The experiments showed that targeting the facet joint with energies of 150–450 J provides controlled and accurate heating at the facet joint edge without penetration to the vertebral body, spinal canal, or root foramina. Treating with reduced diameter of the acoustic beam is recommended since a narrower beam improves access to the targeted areas.

**Conclusions:**

MRgFUS can safely and effectively target and ablate the facet joint. These results are highly significant, given that this is the first study to demonstrate the potential of MRgFUS to treat facet joint pain.

## Introduction

Lumbar zygapophysial joint arthropathy is a challenging condition affecting up to 15% of patients with chronic low back pain. The onset of lumbar facet joint pain is usually insidious with predisposing factors including spondylolisthesis, degenerative disc pathology, and old age [1]. Facet joint ablation to reduce low back pain is a medically accepted and reimbursed procedure, which is gaining more and more popularity recently. The evidence for pain relief after radiofrequency (RF) neurotomy of the cervical and lumbar medial branch nerves is moderate for short- and long-term pain relief, and indeterminate for thoracic facet neurotomy [2,3]. Noninvasive thermal ablation using magnetic resonance-guided focused ultrasound (MRgFUS) has been found to be clinically effective for the palliation of pain caused by bone metastases [4-6] and is currently being evaluated in a pivotal clinical study.

MRgFUS is a well-established technology for the noninvasive thermoablation of various benign and malignant soft tissue and bone tumors. The ExAblate 2000® system (InSightec Ltd., Tirat Carmel, Israel) delivers treatment using focused ultrasound (FUS) in conjunction with real-time magnetic resonance imaging (MRI) guidance to selectively ablate targeted tissue by inducing localized heating [7]. The uniqueness of MRgFUS is based on a technology that facilitates real-time MRI monitoring and control, thereby supplying a high-quality anatomical image of the patient's internal organs and a real-time temperature map for monitoring the thermal rise in the targeted region [7-9]. Accordingly, this allows for treatment adjustments based on ongoing monitoring of the therapeutic effect on the tissue and also assures maximum efficiency and safety. Such real-time feedback does not exist for other localized treatment modalities. The current study evaluated the feasibility, safety, and efficacy of MRgFUS for facet joint pain palliation using a series of *in vitro* and animal studies, as well as a simulated treatment on a healthy human volunteer. Specifically, this study aimed to build the foundation of data required for initiating phase 1 clinical research.

The rationale behind performing several types of experiments was to evaluate the potential of MRgFUS for facet joint ablation using various tools, thereby compensating for the advantages and disadvantages of each. The goal of the phantom experiment was to measure the relative thermal rise in the entire three-dimensional volume of tissue surrounding the vertebra using MR thermometry. The goal of the thermocouple experiments was to provide a more accurate and absolute thermal reading focused on the areas, which if damaged, could cause neurological deficits. The goal of the animal experiments was to examine real-time thermometry in a living organism as well as posttreatment clinical imaging and histopathology evaluation of treatment outcome. The goal of the simulated treatment on a healthy human volunteer was to project our animal study results on a human anatomical model.

## Materials and methods

In order to evaluate the feasibility, safety, and efficacy of MRgFUS for facet joint pain palliation, a phantom experiment, two thermocouple experiments, three *in vivo* pig experiments, and a simulated treatment on a healthy human volunteer were conducted. All experiments were conducted using the ExAblate 2000® System, with imaging done by the system pelvic coil. The experiments took place at Sheba Medical Center (Tel Hashomer, Israel). The research team constituted collaboration between Dr. Sagi Harnof and Dr. Zion Zibly (neurosurgeons), Dr. Yael Inbar (radiologist), Dr. Israel Caspi and Dr. Shay Tenenbaum (orthopedic spine surgeons), and Dr. Itay Goor-Aryeh and Dr. Adrian Greenfeld (anesthesiologists and pain specialists). All animal experiments were performed under local animal ethics committee approvals. The imaging session on a human volunteer was performed with approval from the local human ethics committee (Sheba Medical Center, Israel) and after the subject gave his consent. Based on current results and accumulated data from bone metastases ablation [4,5], we estimated that sonications of 150–450 J with appropriate angling would provide the desired therapeutic effect for accurate ablation of the facet joint within a safe margin.

### Magnetic resonance-guided focused ultrasound technology

MRgFUS treatment commences with a planning phase, whereby the physician co-registers and analyzes the MR and CT images, defines the targeted area for treatment, and prescribes a detailed treatment plan. After the targeted location is defined and confirmed, the parameters are adjusted using the ExAblate software. The treatment is based on multiple sonication spots that accumulate to cover the targeted volume. In combination with real-time guidance, the ExAblate system maneuvers the robotic arm of the ultrasound probe to deliver the acoustic energy to the targeted region. Since the facet joint is a major contributor of pain in patients with lumbar zygapophysial joint arthropathy, ablating the source of pain has the potential to produce lasting pain relief. Since this process also destroys local nerve endings, potential pain palliation is very rapid. During delivery of the FUS energy to each treatment spot, thermal images provide real-time feedback of the treatment location and measure thermal rise, allowing the physician to adjust the treatment parameters for the desired result. Finally, posttreatment contrast imaging is used to confirm the treatment outcome, based on nonperfused volume (NPV), which represents successful ablation.

### Phantom experiment (MR thermometry)

**Figure 1:**
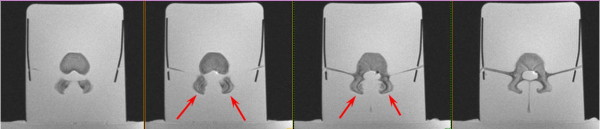
**Axial proton density (PD) images of facet joint phantom used in the experiment.** Red arrows show two facet joints on several slices.

**Figure 2:**
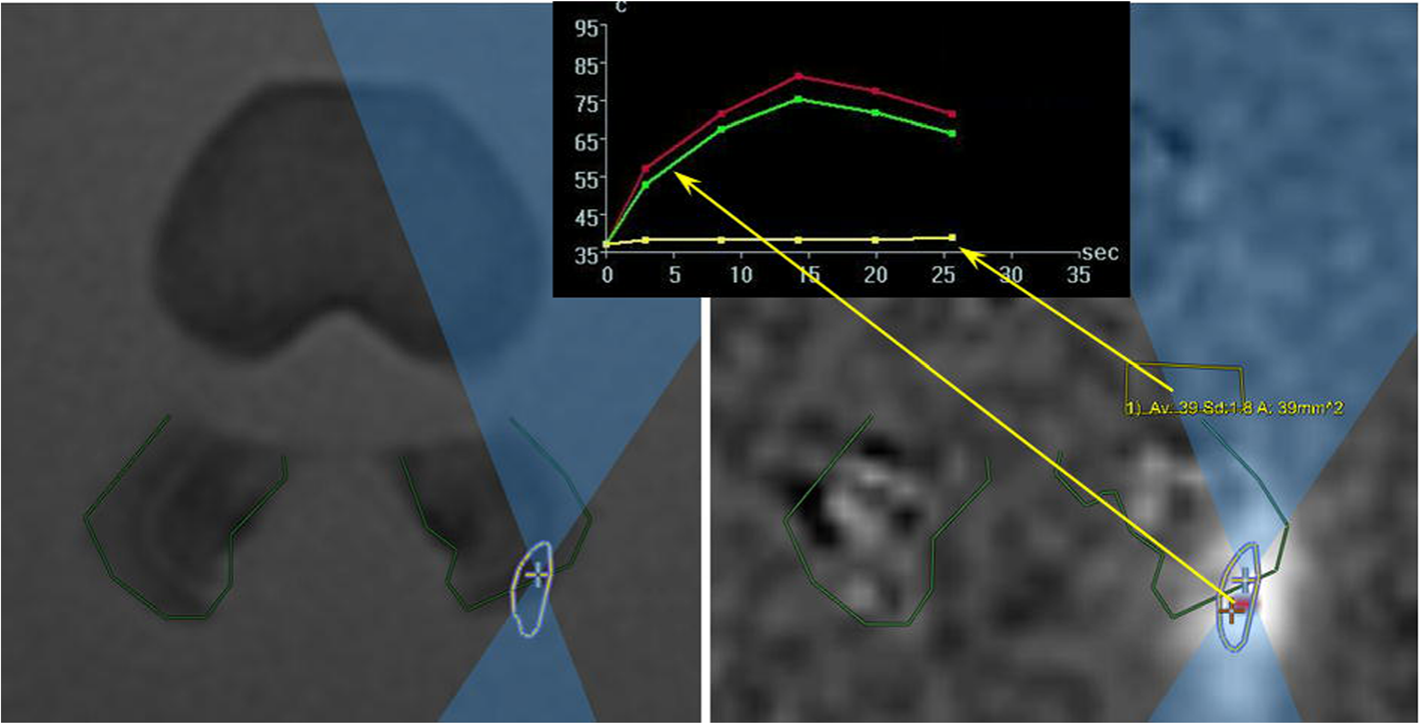
**Temperature measurements at the point of focus and in the far field, adjacent to the intervertebral foramen.** As measured during sonication #13 (900 J). Red (maximum) and green (average) temperature curves represent the point of focus; yellow temperature curve represents the far field, adjacent to the intervertebral foramen.

**Figure 3:**
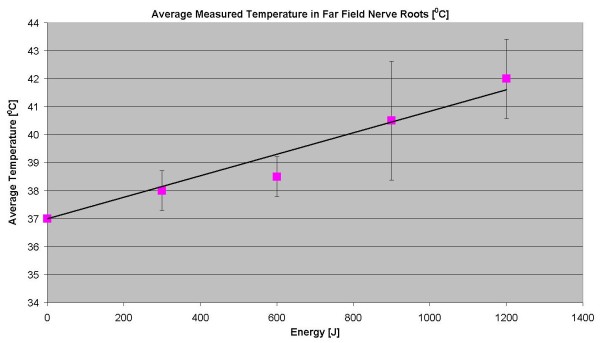
**Thermal rise vs. deposited energy.** The graph shows average temperature measured using MR thermometry in the far field of the acoustic beam, adjacent to the intervertebral foramen as function of delivered energy.

In order to monitor thermal rise in nominal treatment conditions as well as in a worst-case scenario, in the far field of the acoustic beam and near sensitive areas neighboring nontargeted nerves, a phantom experiment was performed. A soft-tissue-mimicking phantom containing two adjacent pig vertebrae was prepared (Figure 1). Two bilateral facet joints were treated using extreme energies (up to four times the expected scenario, Table 1). During the treatment, online monitoring of temperatures was performed using MR thermometry. For each sonication, temperature rise was measured at the focal point and in the far field adjacent to the intervertebral foramen (Figure 2). Measurements from all sonications are summarized and are presented in a single graph showing thermal rise vs. deposited energy (Figure 3).

**Table 1:** Sonication parameters used in the facet joint phantom experiment

**Sonication number**	**Energy (J)**	**Temperature at the focal point (°C)**	**Average temperature in the vicinity of the intervertebral foramen (°C)**
1	300	61	38
2	300	65	38
3	300	73	38
4	300	84	37
5	300	62	38
6	300	59	39
7	300	66	37
8	300	65	38
9	300	71	39
10	600	83	38
11	600	67	37
12	900	72	39
13	900	75	42
14	1,200	78	43
15	1,200	64	41

Summary of the sonication parameters used in the phantom experiment, including acoustic energy delivered, the temperature measured at the focal point on the targeted facet joint, and the temperature measured in the nontargeted intervertebral foramina, located in the far field of the acoustic beam.

### Phantom experiment (thermocouple measurements)

**Figure 4:**
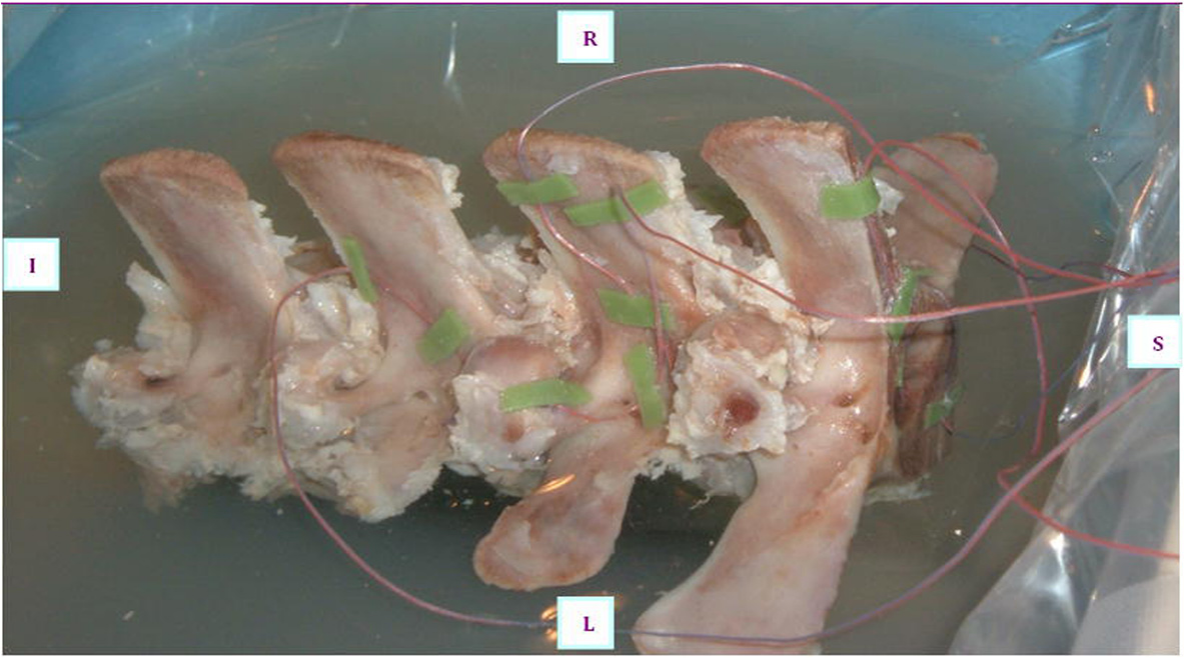
Left view of the pig spine with thermocouples attached adjacent to the intervertebral foramen.

In order to confirm the results of MR thermometry, an experiment using a fresh pig's spine embedded in gel phantom with thermocouples placed in specific areas of the far field was performed (Figure 4). Different sonication parameters and treatment techniques were used in order to assess thermal rise in several far field nerve roots during nominal- and high-energy sonications.

### *In vivo* animal experiments

**Figure 5:**
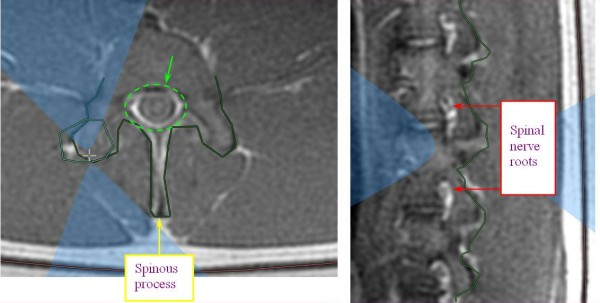
**MR images showing planning of sonication with focal spot on the facet joint.** Appropriate beam orientation is applied in the axial and sagittal planes to protect the spinal nerve roots (white areas on the image, marked with red arrows), the spinous process (marked with yellow arrow), and the spinal canal (marked by green dashed circle) from exposure to the acoustic beam.

**Figure 6:**
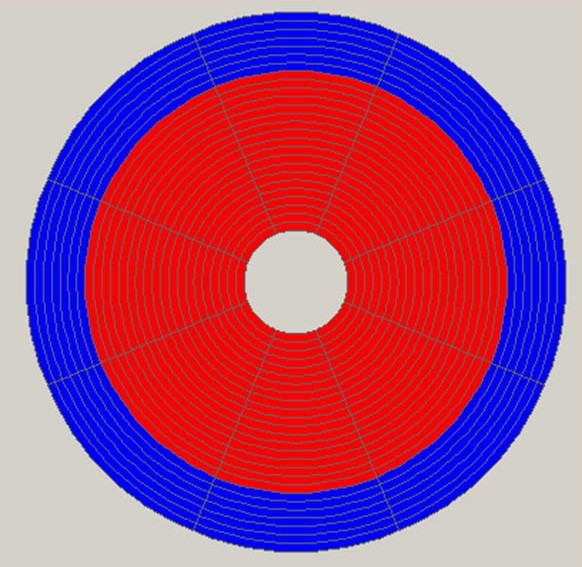
**The ExAblate 2000® transducer and its division to separate elements.** Red represents a working element and blue represents a disabled element. Apodization technique shapes the acoustic beam by switching off certain transducer elements; in this example, seven outer rings of transducer elements were switched off to create a narrower acoustic beam.

**Figure 7:**
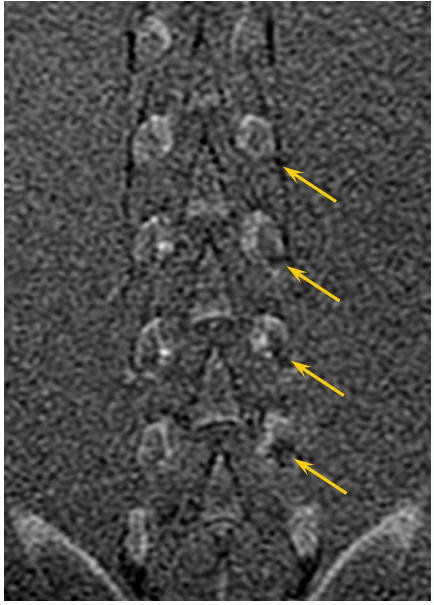
**Coronal T1-weighted contrast-enhanced MR image showing the NPV at the facet joint edges of the treated pig.** More significant treatment effect can be seen on the left facet joints that were treated with 300 J (marked by yellow arrows) vs. the right facet joints that were treated with 150 J.

**Figure 8:**
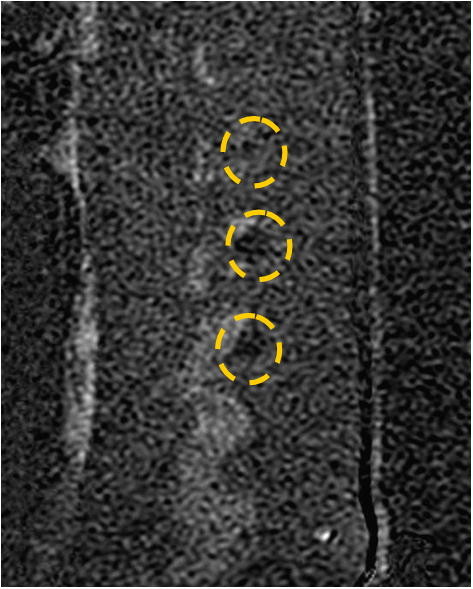
**Sagittal T1-weighted contrast-enhanced subtraction MR image.** The image shows NPV in the left facet joints of the treated pig that were treated with 300 J (marked by yellow circles).

MRgFUS procedures were performed on the facet joints of three young pigs (4–6 months old). Six to eight facet joints in each pig were treated with energies of 150, 300, and 450 J. Thoracic and lumbar spine vertebrae were treated from different orientations with appropriate access. Following the MRgFUS procedure, the second and third pigs were sent to the Animal Research Institute (Lahav, Israel) for veterinary care and follow-up. The first pig remained at the Sheba Animal Laboratory and was imaged and sacrificed after 1 day of follow-up. The second pig went through the same procedure after 1 week of follow-up, and the third pig after 6 weeks.Each facet joint was treated with 2–4 sonications for maximal coverage. Focus was placed on the facet joint surface, and each sonication orientation was adjusted to avoid heating of the spinous process in the near field and nerve roots and spinal canal heating in the far field (Figure 5). In the treatment of the third pig, a transducer apodization technique was used in half of the facet joints that were treated, which allowed shutting off the outer elements of the transducer (blue ring in Figure 6) and sonicating with a narrower beam pass zone (radius = 80%). Planned power was applied using only enabled elements. A narrower beam allows better optimization of the transducer orientation (against the sensitive locations in the beam pass zone) since it minimizes the beam pass zone intersection with the vertebral spinous process.Immediately following the procedure, a set of high-quality images, with and without contrast agent (i.v. gadolinium, 1 ml/5 kg), were acquired to evaluate damage to the targeted tissue (Figures 7 and 8). Upon completion of the follow-up period (1 day, 1 week, and 6 weeks for each of the three pigs, respectively), a dissection was performed to examine the gross pathology of the treated regions and nearby tissue in all three pigs. Specimens of lesions and the nerve roots located anterior to the targeted facet joints were sent to the pathology laboratory (Sheba Medical Center) for histological examination.

### Histology

Histological examination using hematoxylin and eosin stains was performed for all nerve roots located anterior to the treatment area (a total of eight roots). In addition, for each studied animal, hematoxylin and eosin stains were performed for the entire relevant segment of the spinal cord and dura along with two segments below and above the lesion level.

### Treatment simulation on human spine

**Figure 9:**
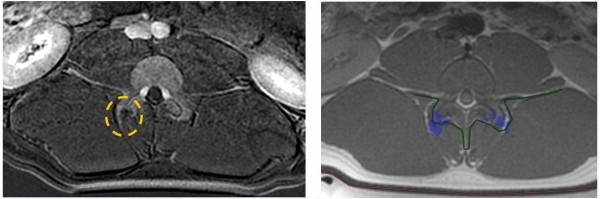
**Facet joint demo model for human treatment.** Axial T2-weighted planning image on the left and sagittal T2-weighted planning image on the right. The blue dashed line demonstrates a narrower beam pass when using apodization technique. This allows better optimization of the transducer orientation since it minimizes the beam pass zone intersection with the vertebral spinous process.

In order to verify safe accessibility of the acoustic beam, a treatment simulation was performed on a human volunteer. The main purpose of this simulation was to check the accessibility of the acoustic beam to human facet joints as well as to test on the human spine different orientation techniques that were applied during pig experiments. During this session, a healthy volunteer was positioned supine, feet first, on a 4-cm gel pad on an ExAblate 2000® table. Images were downloaded later to the demo mode ExAblate 2000® workstation, and a plan of the treatment simulation was generated. These images were taken as part of an imaging session, which was performed in Sheba Medical Center and under approval of the local IRB. An example of a treatment plan for two facet joints is shown in Figure 9.

## Results

### Phantom experiment (MR thermometry)

The phantom experiment showed that targeting the facet joints using the ExAblate 2000® system can produce controlled heating in the facet joint. Spinal nerve roots showed a temperature rise of 6°C in the extreme-case scenario, represented by treatment with 1,200 J, which is four to eight times more than the expected nominal energy (the temperature rise is linearly correlated to the energy). A summary of the measurement results is displayed in Table 1. Although a nominal treatment of the facet joint should be performed using a frequency of *f* = 1.35 MHz in order to minimize the risk of acoustic energy penetration into the vertebra (the higher the frequency, the higher the absorption at the bone surface), in this experiment, several of the nominal and all extreme-case scenario sonications were performed with a frequency of *f* = 1 MHz, for which the penetration of ultrasound into the bone is higher. As recorded in Table 1, there is a linear and consistent elevation of temperature consistent with the increase of energy. The temperature elevation at the focal point did not show this linear and consistent elevation, which is believed to be due to the fact that the focus on the bone surface creates high energy density levels on the facet joint (the smaller the area and the higher the energy levels are), and minor changes like spot location or an angle can create a variety of results.

### Phantom experiment (thermocouple measurements)

The results of the thermocouple experiment confirm the ability to induce a controlled temperature rise at the targeted facet joint and nearby tissue with minimal heating of the nontargeted area. In the extreme-case scenario, which included the worst possible orientation of the acoustic beam, a temperature rise of 5.2°C was measured in the far field at the nerve root location, which is still an acceptable situation. These results correlate well with the results of the MR thermometry phantom experiment.

**Figure 10:**
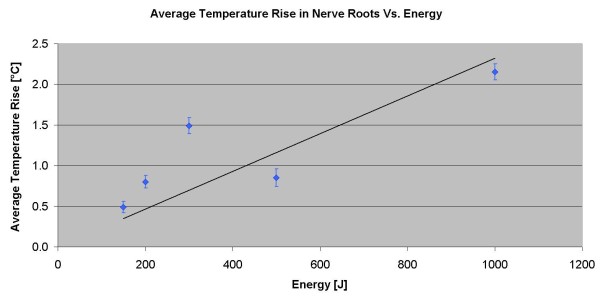
**Average of the temperature rise in the far field of the acoustic beam near the nerve roots.** As measured using a thermocouple.

The results showing specific measurements from the nerve root foramina are summarized in Table 2. In one sonication, using 300 J, the transducer orientation was such that the foramen was at the center of the acoustic beam in the far field. In this case, the temperature rise reached 5.2°C. The measurements are displayed on the graph in Figure 10.

**Table 2:** Summary of the average temperature rise and its range

**Energy (J)**	**Average temperature rise measured (°C)**	**Range (°Ccpa**
150	0.5	0.0–3.0
200	0.8	0.8–0.8
300	1.0	0.2–5.2^a^
500	0.9	0.7–1.0
1,000	2.2	1.0–3.3

Summary of the average temperature measured at the focal point on the targeted facet joint and the temperature measured in the nontargeted intervertebral foramina located in the far field of the sonication according to the acoustic energy delivered. Temperature measurements displayed in this table were acquired using thermocouples. ^a^In one sonication (with an energy of 300 J), the transducer orientation was such that the foramen was at the center of the acoustic beam in the far field. In this case, temperature rise reached 5.2°C.

### *In vivo* animal experiments

**Figure 11:**
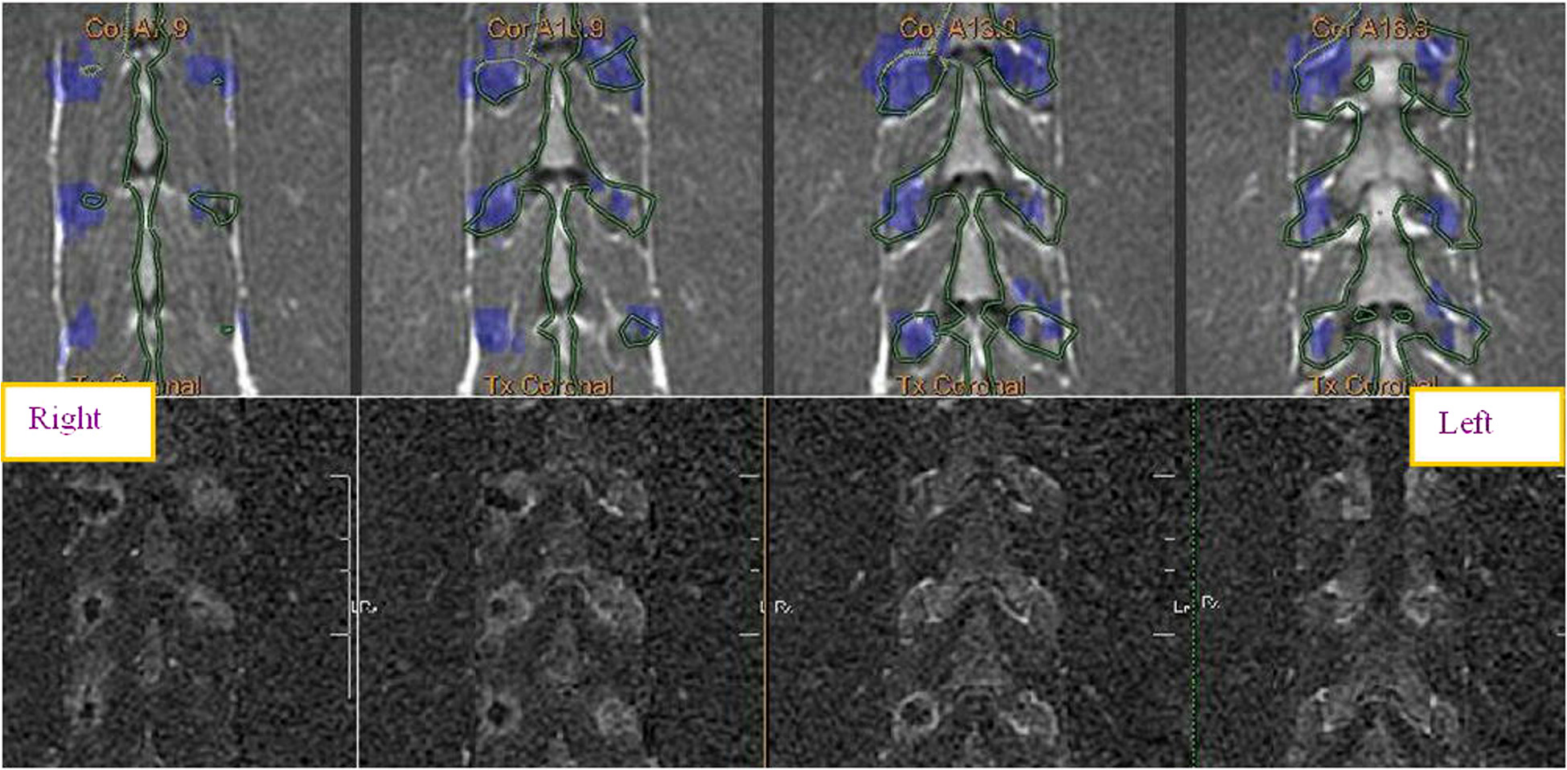
**Posttreatment images of the treated facet joints and treatment effect on facet joints treated with higher energies.** Axial T1-weighted contrast-enhanced MR image (left) showing small nonenhanced area in the right facet joint that was treated with 450 J and soft tissue edema on the left facet joint that was treated with 150 J. This correlates well with the axial PD MR image on the right with blue dose overlay (predicated area of ablation). The lower strip shows multi-slice coronal T1-weighted contrast-enhanced subtraction MR images showing NPV on the right facet joints that were treated with 450 J and soft tissue edema adjacent to the left facet joints that were treated with 150 J, which correlate well with the blue dose overlay on multi-slice coronal PD MR images in the upper strip.

Treating the facet joints with energies of 150, 300, and 450 J resulted in a controlled heating at the facet joint edge with no penetration to the vertebral body, which correlates well to the predicted dose. Posttreatment images showed soft tissue edema and small nonenhanced regions on the edges of the treated facet joints (Figure 11). A higher treatment effect was observed on facet joints that were treated with higher energies (Figure 11).

A neurological follow-up was performed following the experiment, which included a series of tests for walking, light running, abrupt standing up, and eating and drinking habits. According to the veterinary evaluation at Lahav, the last two pigs treated had a normal neurological evaluation result and were eating and drinking normally. In the gross pathology evaluation of the first two pigs (the third still in follow-up), there was no evidence of thermal damage to the spinal column or spinal nerve root.

Based on the final histological evaluation of all specimens, there is no evidence of nerve damage or adverse changes associated with the treatment. No histological changes were seen on the nerve roots or spinal cord; specifically, no ablative changes were seen. However, at the edge of the targeted facet joint, we were able to identify ablative changes as expected.

### Treatment simulation on human spine

We demonstrated the ability to target the facet joint and that the avoidance of critical structures by the ultrasound beam is well within the system's capabilities. The main results of this demo are shown in Figure 9, which shows the system planning spots in green and that the targeted facet joint is device accessible.

## Discussion

Facet joint pain is a common and challenging condition. Current existing treatment options include RF ablation and spinal calm stabilization with hardware. Both these techniques are invasive yet effective with some drawback. The evidence supporting pain relief and control following RF ablation is diverse in the literature, with pain relief success rate of 55%–85% at 1 year [1,2]. Systematic reviews have established moderate and limited evidence for radiofrequency neurotomy of thoracic medial branches [2,3]. Manchikanti et al. reviewed 13 studies and concluded that the evidence for therapeutic benefit is limited for RF neurotomy [10]. In addition, the efficacy of both short- and long-term pain relief is moderate and limited [2]. Stabilization of the effected facet by means of hardware and fusion carries limitation with pain relief and surgical complications, and there is consensus on its value. Cohen et al. reported that there is no evidence to support surgical fusion for lumbar facetal joint pain other than that resulting from traumatic dislocation [1]. Gibson et al. in their review on surgery for degenerative lumbar spondylosis reported that eight trials on instrumented fusion produced higher fusion rates with no improvement in clinical outcomes and higher complication rates. Moreover, it was difficult to assess fusion in the presence of metal work in these trials [11].

A noninvasive, real-time, controlled, safe approach to the treatment of facet joint pain is warranted, for which MRgFUS provides a viable alternative. Utilizing the FUS technique enables tailoring of precise ablation to cover the entire facet joint surface in a single short session, thereby limiting the ablation to the desired location and enhancing the safety profile of the procedure. Furthermore, multiple facet joints can be treated in a single session. The MR guidance provides accurate anatomical visualization for targeting of the facet joint, real-time monitoring, and control of treatment using closed-loop thermal feedback and immediate posttreatment evaluation of outcome using contrast-enhanced imaging.

To enhance the safety of our method, we chose not to target the proximal region of the median nerve but rather to cover the entire facet surface where nerve endings terminate to innervate the joint. By using this approach, we maintained the ablation focus dorsal to the median branch and therefore increased the safety profile of the procedure. Treating the facet joints with energies of 150, 300, and 450 J resulted in a controlled heating at the facet joint edge with no significant penetration to the vertebral body, the spinal canal, or the root foramina. Based on the results and accumulated data from bone metastases ablation [4-6], we estimate that sonications of 150–450 J would provide the desired therapeutic effect with a high safety profile. To ascertain the safety margin, extreme-case scenarios were tested, in some cases using energies of up to 1,200 J, with lower frequencies than would be used during a clinical procedure, (1 MHz instead of 1.35 MHz) with smaller and less calcified vertebrae and in a media with no perfusion or cerebrospinal fluid. Even when considering all of the above, the outcome was still well within the desired safety parameters. Based on the animal studies and human simulation, our results indicate that treating while using apodization is recommended since a narrower beam allows for better optimization of the transducer orientation against the sensitive locations in the beam pass zone. Even in extreme-case scenarios that were tested with extreme energies of up to 1,200 J and the worst orientation, our results showed a maximal temperature rise of 6°C at the spinal nerve roots, which is considered safe (see Table 1). This diminutive increase in temperature following ultrasonic ablation would likely not cause any permanent nerve tissue damage. As shown in other studies, nervous tissue (such as the cranial nerve) present a threshold of 50°C to 60°C to cause permanent damage [12]. In addition, studies done on peripheral nerves (sciatic nerve) showed that permanent damage and conduction block will happen only at a temperature above 50°C [13].

The strength of the current study is that it combines several *in vitro* and *in vivo* studies to test the safety and efficacy of MRgFUS for facet joint ablation. Each experiment compliments the other, thereby compensating for the advantages and disadvantages of each.

The phantom experiment, with the combination of thermal imaging, enables performing volumetric imaging, although the temperature measured is only relative using MRI. The thermocouple experiments generated a more precise and absolute temperature reading but only in limited locations and with decreased spatial certainty due to the artifacts in MRI caused by the thermocouples. The first experiment used thermocouples immersed in water, simulating the nerve root immersed in cerebrospinal fluid. In the second experiment, thermocouples were glued to the bone, again simulating an extreme-case scenario where the nerve is touching the bone. The *in vivo* pig experiment generates an online volumetric thermal reading in an *in vivo* environment, with the same perfusion and type of tissue as in a human subject. However, it involves technical and financial difficulties and has the shortcoming of different vertebral shapes and calcifications (since pigs were young and vertebrae were smaller and less calcified). Nevertheless, this could be an additional safety consideration since it is expected that far field heating in humans would be even lower than anything recorded in the pigs. Human vertebrae are expected to be larger and more calcified and are therefore better at blocking energy. Another safety consideration will be the challenge of planning and targeting the HIFU in humans, where we expect to observe degenerative changes or irregular bony surfaces. This obstacle will be overcome with high-resolution MRI and planning. The benefits of the pig experiments include correlation of the thermal dose with the postprocedural imaging results (T2-weighted and contrast-enhanced MR imaging). It also allows clinical outcome evaluation over the follow-up period for pigs 2 and 3, based on the veterinary exam, as well as gross pathology evaluation of targeted and nontargeted sensitive tissue following the dissection of pigs 1 and 2. The imaging simulation of the healthy human volunteer is advantageous since it shows the human anatomy of the spine. Nevertheless, this is still a healthy volunteer with no back pain due to facet joint problems. As such, human volunteers suffering from facet joint pain should be tested in future clinical trials.

We believe that similar to RF, MRgFUS will be found to be an effective treatment with the advantage of being completely noninvasive and possibly with higher accuracy that may impact clinical outcomes. MRgFUS provides long-term relief using a single noninvasive treatment, eliminating the risk of bleeding and infection, thereby shortening recovery time and reducing morbidity. Since MRgFUS is a radiation-free therapy, there are no long-term toxicity or accumulated dosage effects. As such, repeated treatments can be performed in the same patient as many times as required. MRgFUS has a high safety and efficacy profile and is accurate with supporting data. Other advantages of MRgFUS include its ability to localize the target tissue and monitor accuracy, thermal effects, and treatment outcome in real time, without trajectory constraints, thereby optimizing target coverage.

MRgFUS is already an established treatment for other clinical applications such as uterine fibroid therapy (under FDA and CE approval and the approval of the Ministries of Health in Israel, Japan, and Korea, as well as other regulatory bodies around the world), with an excellent record of safety and efficacy [14-16]. MRgFUS is also under clinical investigation as a treatment for tumors of the breast [17-20], prostate [21], liver [22,23], and brain [24-27]. Given its exemplary safety and efficacy profile, the future of MRgFUS for the treatment of facet joint pain is a bright one. The results of the current study indicate that MRgFUS offers a potentially novel alternative treatment for facet joint pain palliation. Given its noninvasiveness, accuracy, safety, efficacy, and the ability to be performed on an outpatient basis, it may offer a revolutionary solution to patients suffering from facet joint pain. Although further preclinical and clinical studies are required, the current results are promising and provide a solid basis for further exploration.

## Conclusion

In conclusion, our results cumulatively show that MRgFUS can safely and effectively target and ablate the facet joint. This is the first study to demonstrate the potential of MRgFUS to treat facet joint pain and may represent a breakthrough in the treatment of chronic lower back pain due to lumbar zygapophysial joint arthropathy.

## Competing interests

The authors declare that they have no competing interests.

## Authors’ contributions

SH and ZZ preformed the treatment planning and animal studies and drafted the manuscript. YI performed and analyzed the imaging studies. IC and SH collaborated and assisted in the treatment planning. IGA and AH assisted with treatment planning and evaluation. All authors read and approved the final manuscript.
